# Cannabidiol Ameliorates Blood–Brain Barrier Dysfunction and Inhibits Memory Astrocytes Activation in Mice With Sickness‐Like Behaviors

**DOI:** 10.1002/cns.71168

**Published:** 2026-09-16

**Authors:** Yue Chen, Hui Ma, Qian Long, Xuan Fang, Xiaotong Li, Wenhui Xiao, Xinzhou Deng, Fuze Du, Yifei Tan, Yan Wang, Xiaoxia Jiang, Bo Gao, Xin Qiao

**Affiliations:** ^1^ Academy of Military Medical Sciences, Academy of Military Sciences Beijing China; ^2^ Chinese PLA Medical School Beijing China

**Keywords:** BBB, CBD, chronic depression, memory astrocytes, neuroinflammation

## Abstract

**Background:**

Blood–brain barrier (BBB) dysfunction has been increasingly implicated in the pathophysiology of depression; however, effective therapeutic strategies targeting this pathology remain limited. This study aimed to investigate the effect of cannabidiol (CBD) on improving depressive‐like behaviors and BBB impairment, as well as its underlying mechanism.

**Methods:**

Depressive‐like behaviors in mice were assessed via exploratory and despair‐like tests. BBB integrity was evaluated by examining tight junction protein expression in endothelial cells and AQP4 reduction from the perivascular membrane. Astrocyte activation and neuroinflammation were also measured. The therapeutic effects of a single CBD dose were examined.

**Results:**

Mice with depressive‐like phenotypes showed reduced exploratory behavior and increased despair‐like behavior. In the BBB, the expression of tight junction proteins in endothelial cells was downregulated; in astrocytes, decreased AQP4 expression was observed, abnormal activation was enhanced, and neuroinflammation was elevated. A single dose of CBD alleviated all the above pathological changes. Further studies demonstrated that CBD exerted its effects by inhibiting memory astrocyte–associated secondary neuroinflammation, thereby alleviating depressive‐like behaviors in mice with depressive‐like phenotypes.

**Conclusion:**

Experimental results show that CBD treatment can improve BBB dysfunction in depression and further indicates that targeted inhibition of memory astrocytes‐mediated neuroinflammation may promote the treatment of depression.

## Graphical abstract

Depressive‐like mice showed blood–brain barrier disruption with loss of endothelial tight junction proteins, astrocytic AQP4 depolarization, and elevated neuroinflammation. A single dose of cannabidiol restored BBB integrity and astrocyte polarity, suppressed memory astrocyte–mediated secondary neuroinflammation, and alleviated depressive‐like behaviors, suggesting a potential therapy for depression‐associated BBB dysfunction.

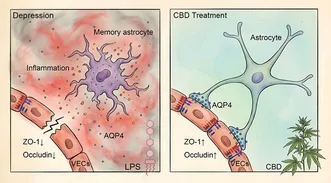

## 1 Introduction

Depression is a prevalent category of mental disorders characterized by core features such as anhedonia, diminished motivation, and stress‐related behavioral abnormalities [1]. These symptoms often manifest as quantifiable behavioral alterations, including reduced exploratory behavior, enhanced avoidance, and increased despair‐like behaviors [2]. However, effective clinical treatments remain lacking. Existing therapies primarily focus on traditional medications and physical methods, which suffer from side effects such as slow onset and short‐term memory impairment [3, 4]. The neuroinflammatory hypothesis posits that aberrant activation of the peripheral inflammatory response can influence central nervous system function through multiple pathways, representing a significant pathological foundation of depression. Peripheral inflammatory signals, mediated via humoral and neural pathways, can compromise blood–brain barrier (BBB) homeostasis, thereby exacerbating central neuroinflammation and contributing to the development of depression‐related behavioral and cognitive dysfunctions [5, 6, 7]. Thus, identifying highly effective and safe drugs for BBB injury in depression and exploring their underlying mechanisms are of great importance.

Cannabidiol (CBD), a natural extract from cannabis, possesses multiple biological functions including cardioprotection, anti‐inflammation, anti‐cancer, anti‐oxidation, and neuroprotection [8]. Additionally, it plays indispensable roles in relieving depression and regulating BBB function [9]. CBD ameliorates depression by upregulating brain‐derived neurotrophic factor, inhibiting neuroinflammation [10], alleviating oxidative stress [11], and modulating neurotransmitter transmission [12]. However, its protective mechanism against depression and BBB impairment requires further investigation.

Increasing evidence suggests that CBD can protect the BBB. As vital components of the BBB, endothelial cells and astrocytes play complex yet crucial roles in regulating BBB integrity and permeability [13]. Loss of tight junction proteins in endothelial cells and abnormal activation of astrocytes may trigger neuroinflammation and brain homeostasis imbalance, causing severe tissue and organ damage [14, 15]. Memory astrocytes, an epigenetically controlled subset driven by ACLY‐ and p300‐dependent histone acetylation, exhibit faster and stronger pro‐inflammatory responses upon restimulation following an initial stimulus [16]. During the pathological process of depression, memory astrocytes may contribute to neuroinflammation and BBB dysfunction. However, direct and systematic evidence regarding the interaction between memory astrocytes and the BBB in depression is still lacking. Furthermore, whether CBD can ameliorate BBB function and promote depressive recovery by targeting memory astrocytes remains unclear.

This study aims to investigate the effects of CBD on depression and the underlying mechanisms, with a particular focus on the role of memory astrocytes in the BBB in mediating the effects of CBD, thereby providing new potential targets for depression therapy.

## 2 Materials and Methods

### 2.1 Animals and Modeling

The 7‐week‐old male C57BL/6J mouse was purchased from Spiff (Beijing). All mice were housed in standard animal facilities with controlled temperature (21°C) and photoperiod (12 h light/12 h darkness), free water and food. All animal experiments were conducted in accordance with the “Guidelines for the Care and Use of Experimental Animals” approved by the Beijing Institute of Basic Medical Sciences. All experimental protocols are approved by the ethical Review Committee of animal testing institutions.

LPS‐induced mouse model: To establish a chronic inflammation‐associated depression model, male C57BL/6J mice were intraperitoneally injected with LPS (1 mg/kg in sterile 0.9% saline) once daily for seven consecutive days between 09:00 and 09:30 a.m. [17]. Control mice received equivalent volumes of saline at the same time points.

Drug administration: CTB was administered intraperitoneally (0.5 mg/kg in 2% Tween 80 saline) 30 min after LPS injection on Day 7. CBD treatment (30 mg/kg, single i.p. injection in 2% Tween 80 saline) was given on Day 8 following the 7‐day LPS regimen [18]. Corresponding vehicle injections (2% Tween 80 in saline) were applied to LPS model, CTB intervention, and control groups to serve as solvent controls.

### 2.2 Behavior Assay

#### 2.2.1 Open Field Test (OFT)

OFT was used to supplement the anxiolytic effects evaluation. Following 1 h of habituation under experimental conditions, mice were placed in a 40 cm × 40 cm × 40 cm field for 5 min to freely explore. Behavior was monitored and tracked using the Labmaze Behavioral Analysis System (version 3.0, Beijing Zhongshidi Chuang Technology Development Co. Ltd.), an adjacent 20 cm × 20 cm area defined as the middle with the remaining zone along the walls defined as the periphery. Cumulative time and trajectories spent in each area were compared between groups.

#### 2.2.2 Elevated Plus Maze (EPM)

Anxiety‐like behaviors were evaluated with the EPM. Following 1 h of habituation under experimental conditions, mice were placed in a cross‐shaped maze for 5 min. The maze supports two sets of arms (10 cm width × 50 cm length, open‐without walls and closed arms with 50‐cm high wall) 1 m above floor level (Beijing Zhongshidi Chuang Technology Development Co. Ltd.). Cumulative time and trajectories spent in open arms were compared between groups.

#### 2.2.3 Tail Suspension Test (TST)

To gain more insight into depression‐like behavior, TST was applied [19]. Mice were habituated to the room for 1 h before testing. Next, mice were suspended by the tail 1–2 mm from the tail base for 6 min, with the first minute for adaptation, and their behavior was videotaped. Automated tracking recorded the active state, immobility time, and struggle frequency during the last 5 min.

#### 2.2.4 Forced Swimming Test (FST)

To evaluate helplessness, FST was used [14]. Mice were habituated to the room for 1 h before testing. Mice were then placed into a transparent cylinder (20 cm diameter, 30 cm height) filled with water (15–20 cm deep, 22°C–25°C) and videotaped for 6 min. The first minute was an adaptation period, followed by recording of active state, immobility time, and struggle frequency.

### 2.3 Immunofluorescence Staining

Following fixation in 4% paraformaldehyde, brain sections or cell slides underwent permeabilization and blocking with PBS containing 10% donkey serum and 0.1% Triton X‐100 for 1 h. Primary antibodies were incubated overnight at 4°C. After PBS washes, secondary antibodies were applied for 1 h at room temperature, followed by DAPI (Thermo Fisher, D1306) staining and mounting. The primary antibodies were anti‐AQP4 (Proteintech, 16473‐1‐AP, Rabbit), anti‐CD31 (Proteintech, 11265‐1‐AP, Rabbit), anti‐occludin (Invitrogen, 3F10, Mouse), anti‐ZO‐1 (Invitrogen, 61‐7300, Rabbit), anti‐NANOG (Proteintech, 14295‐1‐AP, Rabbit), anti‐GFAP (Servicebio, GB11096, Rabbit), anti‐ACLY (ProteinTech Group, 15421‐1‐AP, Rabbit), anti‐p300 (Cell Signaling Technology, 70088S, Rabbit). The secondary antibodies were Cy3 (Jackson ImmunoResearch, 711‐165‐152) and Alexa 488 (Jackson ImmunoResearch, 715‐546‐150).

### 2.4 Western Blot

The hippocampus of each group of mice was extracted and protein samples were prepared by adding RIPA lysis buffer. The BCA protein detection kit (Thermo Fisher, 23255) was used to determine the protein concentration. The target protein was isolated using 8% Tris gels via SDS‐PAGE, then imprinted on PVDF membrane and incubated overnight with the antibody to be detected. After the film was washed with TBST, the secondary antibodies of the corresponding species were incubated at room temperature for 1 h. The labeled proteins were visualized using the ECL chemiluminescence detection kit (Merck Millipore, 34075). The primary antibodies were anti‐β‐actin (Cell Signaling Technology, 3700S, A19056, mouse, 1:1000), anti‐occludin (Invitrogen, 3F10, Mouse, 1:1000), and anti‐ZO‐1 (Invitrogen, 61‐7300, Rabbit, 1:1000).

### 2.5 Cell Culture

hCMEC/D3 cells and HT22 cells were purchased from Procell, and U87MG cells were obtained from the Chinese Academy of Medical Sciences. hCMEC/D3 cells were cultured in endothelial cell medium (ECM), while U87MG cells and HT22 cells were cultured in Dulbecco's Modified Eagle Medium (DMEM). Both media were supplemented with 10% fetal bovine serum (FBS; Gibco, MA, USA) and 1% penicillin/streptomycin (100 U/mL; Gibco, MA, USA). Cells were maintained at 37°C in a humidified 5% CO_2_ incubator. The culture medium was refreshed three times weekly.

### 2.6 Establishment of an In Vitro Blood–Brain Barrier (BBB) Model

The BBB co‐culture model in vitro is an experimental method to simulate the interaction and microenvironment between the BBB component cells. The in vitro BBB model was divided into four groups, with hCMEC/D3 cells seeded at a density of 1 × 10^5^ cells/cm^2^ on the apical side of Transwell inserts in all groups, and U87MG cells seeded at a density of 4 × 10^4^ cells/cm^2^ with different configurations: the monoculture model only included hCMEC/D3 cells seeded on the Transwell inserts; the direct‐contact co‐culture model involved co‐seeding of U87MG cells and hCMEC/D3 cells on the apical side of the Transwell inserts; the indirect‐contact co‐culture model had U87MG cells seeded on the basolateral side of the Transwell membrane; and the non‐contact co‐culture model was established by seeding U87MG cells on the bottom of the well plate with Transwell inserts containing hCMEC/D3 cells placed above.

### 2.7 iPSCs Induced Differentiation Into iPSC‐hBMEC

The induction protocol of iPSCs (Stem Cell Bank, Chinese Academy of Sciences, DYR0100) was derived from an endothelial differentiation kit (STEMCELL, 08005), and the induction cycle was 7 days. On Day 0, iPSCs were prepared into a single‐cell suspension and inoculated into a 6‐well plate coated with matrigel (Corning, 354,277) at a density of 7.5 × 10^4^/cm^2^, and mTeSR1 medium +10 μM Y‐27632 was added. On Days 1 to 2, the medium was replaced with mesoderm induction medium and the liquid was changed every day. On Days 3 to 6, the endothelial cell induction medium was changed every 2 days. On Day 7, the induced endothelial cells were cultured or passed.

### 2.8 Resistivity Detection

The TEER of the in vitro BBB model under various treatments was determined following previously described protocols with minor adjustments [20]. The model was cultured until the electrical resistance reached its peak. Subsequently, the model was administered vehicle (control), LPS (1 μg/mL), or LPS combined with CBD (10 μmol/L) for 24 h. TEER measurements were performed using an epithelial voltmeter (RE1600, Beijing DongGong Technology) equipped with a hands‐free electrode, which was sterilized using 75% alcohol and ultraviolet irradiation prior to each use. All TEER values were calculated after subtracting the background resistance of blank Transwell inserts (containing medium only). TEER (Ω·cm^2^) was calculated using the following formula: (measured value − blank control value) × effective membrane area. All experimental data were processed using this method.

### 2.9 Detection of Drug Membrane Permeability in Positive Quality Control

#### 2.9.1 Preparation of Positive Controls

Weigh out an appropriate amount of positive controls (Atenolol, Metoprolol, Digoxin, Selleckchem), and dissolve them in DMSO to prepare a mixed stock solution of 200 μM. Dilute the working solution to 2 μM using HBSS/Hepes/0.1% BSA buffer solution.

#### 2.9.2 Bidirectional Transport Experiments

Discard the Transwell apical and basolateral media, wash the cells three times with prewarmed HBSS/Hepes/0.1% BSA (100 μL in the upper chamber, 600 μL in the lower chamber) at 37°C for 30 min. Discard the buffer solution, and for the A‐B direction transport experiment: add 150 μL of the positive control working solution to the upper chamber as the donor compartment, immediately collect 50 μL into a 1.5 mL EP tube as sample D0; add 600 μL of blank HBSS/Hepes/1% BSA to the lower chamber as the acceptor compartment. For the B‐A direction transport experiment: add 650 μL of the positive control working solution to the lower chamber as the donor compartment, immediately collect 50 μL into a 1.5 mL EP tube as sample D0; add 100 μL of blank HBSS/Hepes/1% BSA to the upper chamber as the acceptor compartment. Incubate in a 37°C, 5% CO_2_ incubator for 2 h, collect 100 μL samples from both the donor and acceptor chambers at the end of the incubation period, temporarily store all samples at −40°C for LC–MS/MS analysis.

#### 2.9.3 LC–MS/MS Detection Conditions for Positive Controls

Chromatographic conditions: Phenomenex C18 column (3.0 mm × 50 mm, 2.6 μm): Mobile phase: water (A)‐acetonitrile (B) containing 0.1% formic acid; gradient elution as follows: (0.0–0.3 min, 1% B; 0.3–1.5 min, 1%–90% B; 1.5–3.5 min, 90% B; 3.5–5.0 min, 1% B). Column temperature 40°C, injection volume 5 μL.

Mass spectrometric conditions: Electrospray ion source (ESI), Multiple Reaction Monitoring (MRM) in positive ion mode, collision gas (CAD) 8 psi; curtain gas (CUR) 35 psi; ion spray voltage 5500 V; ion source temperature 550°C. Detailed parameters are shown in the Table 1.

**TABLE 1:** Mass spectrometry detection conditions for positive quality control drugs and propranolol (internal standard).

Compound	MRM	*m*/*z*		CE	DP	CXP
		Precursor Ion	Product Ion	(eV)	(eV)	(eV)
Atenolol	+	267.0	145.0	50.0	76.0	18.0
Metoprolol	+	268.1	116.1	30.0	140.0	11.0
Digoxin	+	798.5	97.2	55.0	190.0	20.0
Propranolol	+	260.1	116.1	30.0	100.0	10.0

### 2.10 Quantitative RT‐PCR

Total RNA from cells was collected by TRIzol (Invitrogen). Total RNA was extracted by chloroform extraction and isopropanol precipitation according to the manufacturer's recommendations. Reverse transcription was performed using 5 × RT Master Mix (Toyobo, 037400). Quantitative PCR was performed with 2 × T5 Fast qPCR Mix (SYBR) (TSINGKE, TSE202). Each amplification cycle consisted of an initial step at 95°C (5 min), followed by 40 cycles of denaturation at 95°C for 15 s, annealing at 60°C for 1 min, and extension at 72°C for 30 s [21]. β‐actin was used as an internal control. The primer sequences used are shown in the Table 2.

**TABLE 2:** Primer sequences for qRT‐PCR of each target gene.

Target gene	Forward primer (5′‐3′)	Reverse primer (5′‐3′)
*β‐Actin*	GGCTGTATTCCCCTCCATCG	CCAGGTAACAATGCCATG
*Occludin*	AGCTTCCATTAACTTCGCCTGTG	TCGCCGCCAGTTGTGTAGTC
*Zo‐1*	GATGTTTATGCGGACGGTGG	CATTGCTGTGCTCTTAGCGG
*Nanog*	AGAACTCTCCAACATCCTGAACCTC	CCTGCGTCACACCATTGCTATTC
*Pousf1*	GAGAACCGAGTGAGAGGCAACC	CTGGGCGATGTGGCTGATCTG
*Sox2*	GCCCAGGAGAACCCCAAGATG	GCAGCCGCTTAGCCTCGTC
*Shh*	GCGAGATGTCTGCTGCTAGTCC	TGCCTCCTCTTCCCGAACCC
*Fgf8*	CCGCAAGGGCTCCAAGACG	GGCTCTGCTCGGTGGTGTG
*Tnf‐α*	GGACTAGCCAGGAGGGAGAACAG	CCAGTGAGTGAAAGGGACAGAAC
*Il‐1β*	TCGCAGCAGCACATCAACAAGAG	AGGTCCACGGGAAGACACAGG
*Il‐6*	TCTGGAGCCCACCAAGAACGATAG	GTCACCAGCATCAGTCCCAAGAAG
*Ccl2*	ACTGCATCTGCCCTAAGGTCTTC	TCACTGTCACACTGGTCACTCC
*Nos2*	GCACCACCCTCCTCGTTCAG	GACAATCCACAACTCGCTCCAAG
*Gfap*	GCCACCAGTAACATGCAAGA	GGCGATAGTCGTTAGCTTCG
*Ep300*	TCCGCCTCATCGCTTGTCC	AGAGAACTCCAGGTGCTTGTCC
*Acly*	TGATGGGAGAAGTTGGGAAGACC	AGGAGGAAGTTGGCAGTGTGAG
*Cd31*	AAGGTGGTGGAGTCTGGAGAGG	CTGGGTGGCATTTGAGGTCATTTG

### 2.11 Elisa

Cell supernatants were collected from each experimental group for subsequent analysis. The concentrations of TNF‐α, IL‐1β, and IL‐6 in the supernatants were quantified using commercial ELISA kits strictly following the manufacturer's instructions. The optical density (OD) of each well was measured at 450 nm with a microplate reader, and a standard curve was generated to calibrate the protein concentrations. The final levels of TNF‐α, IL‐1β, and IL‐6 in each group were determined by extrapolating the measured OD values against the standard curve. All assays were performed with technical replicates to ensure the reliability and reproducibility of the data.

### 2.12 Neuronal Viability Assay

Ten thousand HT22 cells were seeded into each well of 96‐well plates. After thorough washing with 1× PBS, the medium was replaced, and 200 μL of astrocyte‐conditioned medium was added to each well. Eighteen to twenty‐four hours later, the supernatant was harvested. Cell viability of HT22 cells was assessed by measuring LDH release using the CytoTox 96 non‐radioactive cytotoxicity assay kit (Promega, G1780) following the manufacturer's recommended protocol.

### 2.13 RNA Sequencing (RNA‐Seq) Analysis

Cells were collected from each experimental group for subsequent analysis. Total RNA was extracted from the cells using TRIzol reagent (Invitrogen, 10,296,010) according to the manufacturer's protocol. RNA purity and quantification were evaluated using a NanoDrop 2000 spectrophotometer (Thermo Scientific, USA). RNA integrity was assessed using the Agilent 2100 Bioanalyzer (Agilent Technologies, Santa Clara, CA, USA). Then, the libraries were constructed using the TruSeq Stranded mRNA LT Sample Prep Kit (Illumina, San Diego, CA, USA) according to the manufacturer's instructions. Transcriptome sequencing and analysis were conducted by Majorbio Co. Ltd. (Shanghai, China). The libraries were sequenced on an Illumina HiSeqX Ten platform, and 150 bp paired‐end reads were generated. Raw data (raw reads) in fastq format were first processed using Trimmomatic, and the low‐quality reads were removed to obtain clean reads. The thresholds for significantly differential expression were set to *p* < 0.05 and |log2 (fold change)| ≥ 1.

### 2.14 Assessment of BBB Permeability Using FITC‐Dextran

BBB permeability was evaluated by measuring extravasation of fluorescein isothiocyanate (FITC)‐conjugated dextran (4 kDa, FD4, Sigma‐Aldrich). Two hours before sacrifice, mice were injected intravenously via the tail vein with FITC‐dextran (25 mg/mL in sterile PBS, 50 μL per mouse).

### 2.15 Statistical Analysis

Data are presented as the mean ± SEM. Two‐way ANOVA was used to analyze all behavioral tests between and among the treatment groups. In anatomical and biochemical studies, one‐way or two‐way ANOVA was used to compare multiple groups. A Bonferroni post hoc analysis was used to determine whether differences were significant. The differences between two groups were tested with the two‐tailed Student's *t*‐test. The criteria for statistical significance were *p* < 0.05 [22].

## 3 Results

### 3.1 CBD Alleviates Anxiety and Depressive‐Like Behavior in Mice

**FIGURE 1:**
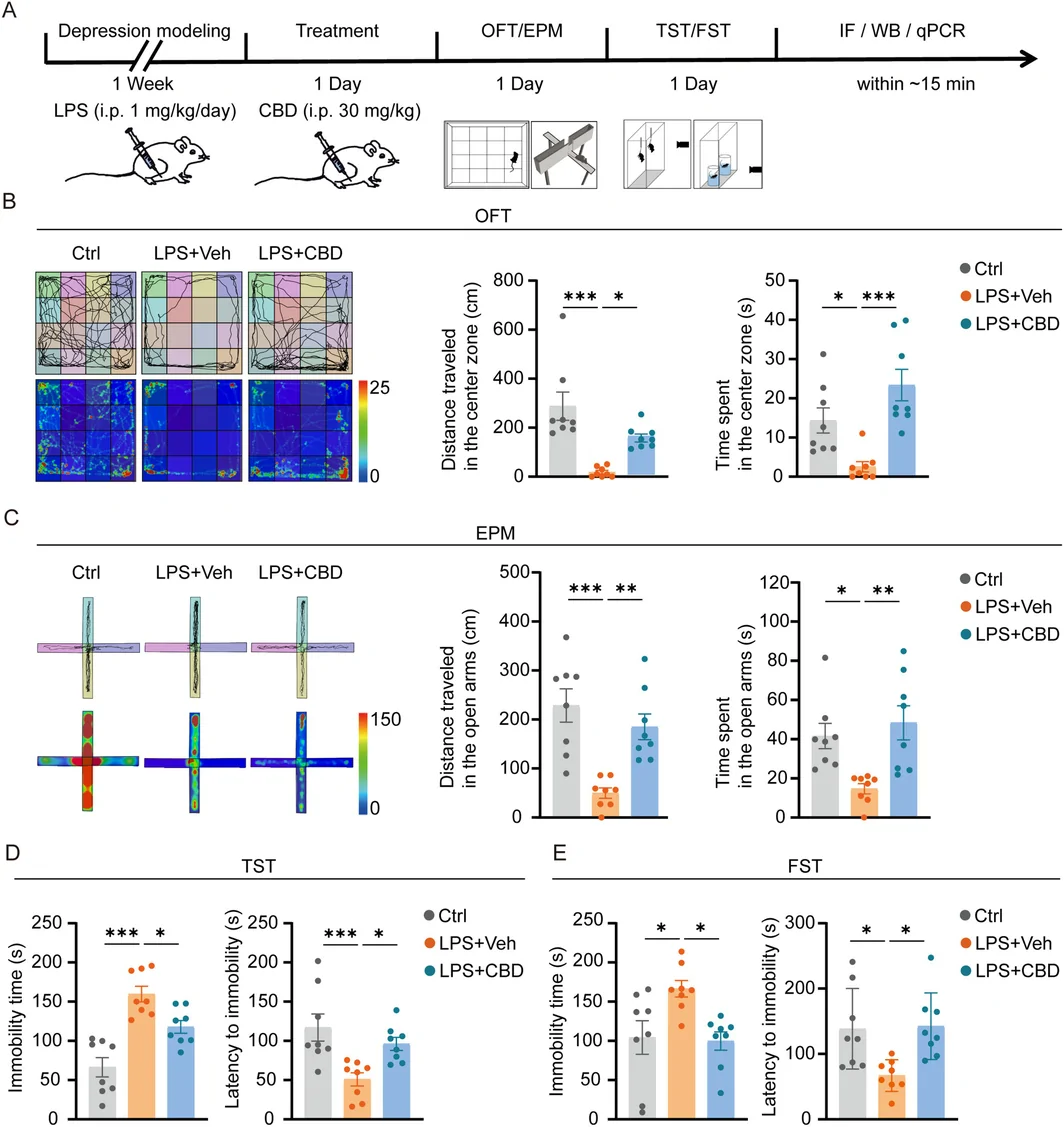
CBD alleviates anxiety and depressive‐like behaviors in mice. (A) Schematic timeline of the experimental design. Male C57BL/6J mice were randomly assigned to three groups: Control, LPS, and LPS + CBD. All mice received intraperitoneal (i.p.) injections for seven consecutive days. The Control group received an equal volume of saline, while the LPS and LPS + CBD groups received LPS (1 mg/kg in saline). On Day 8, the LPS + CBD group was administered a single injection of CBD (30 mg/kg in saline containing 2% Tween 80), whereas the Control and LPS groups received an equivalent volume of the CBD vehicle. Behavioral tests commenced 24 h post‐CBD injection. Open field test (OFT) and elevated plus maze (EPM) were conducted sequentially on the first day, followed by tail suspension test (TST) and forced swim test (FST) on the second day. After completing the TST and FST, each mouse was immediately euthanized within ~15 min. Brain tissues were rapidly dissected for subsequent immunofluorescence analysis, Western blot and qRT‐PCR assays. Immunofluorescence (IF) was performed to assess the expression of ZO‐1, CD31, AQP4, and GFAP; western blot (WB) was used to quantify ZO‐1, occludin, and β‐Actin; and qPCR measured the mRNA levels of TNF‐α, IL‐1β, CCL2, IL‐6, NOS2, and GFAP. (B) Center distance and center time in the OFT. (C) Open arm distance and open arm time in the EPM. (D) Immobility time and latency to immobility in the TST. Data are presented as mean ± SEM (*n* = 8). One‐way ANOVA with Tukey's post hoc test was used for statistical analysis; ns, not significant; *p < 0.05, **p < 0.01, ***p < 0.001. (E) Immobility time and latency to immobility in the FST. Data are presented as mean ± SEM (*n* = 8). One‐way ANOVA with Tukey's post hoc test was used for statistical analysis; ns, not significant; *p < 0.05, **p < 0.01, ***p < 0.001.

Neuroinflammation is thought to be fundamental in the etiology of MDD. Hence, we exposed male C57BL/6 mice to 7 days of lipopolysaccharide (LPS) intraperitoneal injection to mimic depression, administered a single acute dose of CBD (30 mg/kg, i.p.) after LPS, and then evaluated its effects (Figure 1A). To ensure that the behavioral changes were primarily driven by emotional alterations rather than motor deficits, we measured the total distance traveled in the open field test and monitored body weight throughout the 7‐day modeling period. The results showed that LPS injection reduced locomotor activity in both the LPS + Veh and LPS + CBD groups. However, there was no significant difference in the total distance traveled between the LPS + Veh and LPS + CBD groups, whereas the LPS + CBD group exhibited a greater distance traveled in the center zone compared to the LPS + Veh group, and their body weight decreased transiently in the early stage of modeling and then gradually recovered with an increasing trend in the later stage. These findings indicate that the model mice retained sufficient locomotor capacity, and the reduction in center zone distance is unlikely to result from motor deficits; rather, it more likely reflects anxiety−/depressive‐like behavioral changes (Figure S1A,B). The mice in the LPS group exhibited a reduced central distance and time in the OFT (Figure 1B) and a reduced open arm distance and time in the EPM (Figure 1C). These results suggested decreased anxiety after LPS. The LPS exposure led to increase of depression‐related behaviors as indicated by longer immobility time and shorter latency in the TST (Figure 1D) and FST (Figure 1E) compared to the control group. Furthermore, we observed significant amelioration of these phenotypes in mice that were treated with a dose of CBD. These results indicated that the anxiolytic and antidepressant effects of CBD.

### 3.2 CBD Restores the Expression of Tight Junction Proteins in BBB Endothelial Cells in LPS‐Induced Models

**FIGURE 2:**
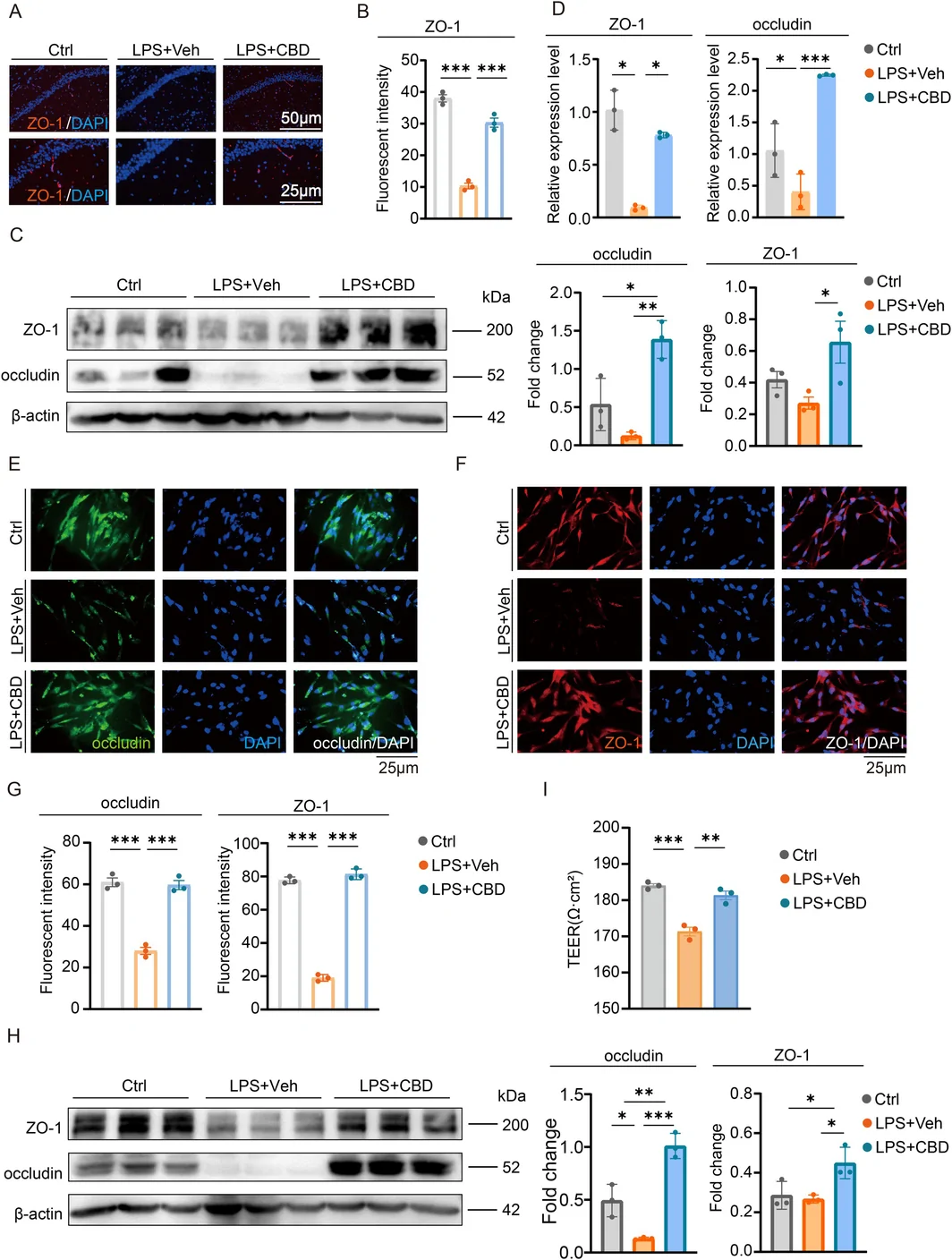
CBD restores the expression of tight junction proteins in BBB endothelial cells in LPS‐induced models. (A) Immunofluorescence staining of ZO‐1 in the hippocampal region. (B) Quantification of ZO‐1 immunofluorescence intensity shown in panel A. (C) Western blot analysis and quantification of ZO‐1 and occludin protein expression in hippocampal tissue. (D) qRT‐PCR analysis of ZO‐1 and occludin mRNA expression in the hippocampus. (E, F) Immunofluorescence staining of occludin (E) and ZO‐1 (F) in hCMEC/D3 cells. (G) Quantification of immunofluorescence intensity shown in panels (E and F). (H) Western blot analysis and quantification of protein expression in hCMEC/D3 cells from the three groups. (I) Transendothelial electrical resistance (TEER) measurements of hCMEC/D3 monolayers. Data are presented as mean ± SEM (*n* = 3). One‐way ANOVA with Tukey's post hoc test was used for statistical analysis; ns, not significant; *p < 0.05, **p < 0.01, ***p < 0.001.

Since BBB dysfunction plays an important role in depression, we focused on investigating the protective effects of CBD on it. A more sensitive FITC‐dextran (4 kDa) assessment revealed significant extravasation in LPS‐treated mice attenuated by CBD treatment while control mice showed almost no leakage (Figure S1D). Given that depression‐associated BBB changes are often more subtle and involve molecular alterations at the neurovascular interface, we proceeded to conduct further cellular and molecular analyses to assess BBB integrity at higher resolution. The integrity and permeability of the BBB is due mainly to the tight junctions between endothelial cells, which are composed of the proteins zonula occludens‐1 (ZO‐1) and occludin [23]. Thus, we conducted western blotting analysis, qRT‐PCR analysis and immunofluorescence staining analysis and found that CBD recovered the expression of ZO‐1 and occludin in both the hippocampus of mice subjected to depression (Figure 2A–D; Figure S1E) and hCMEC/D3 cells exposed to LPS (Figure 2E–H). The TEER values depict a functionally compact layer of cells that resist permeability [24]. Next, we found that CBD reversed the increase in hCMEC/D3 monolayer permeability induced by LPS, as evidenced by the transendothelial electrical resistance (TEER) (Figure 2I). Collectively, these data suggested that CBD promoted the recovery of BBB integrity.

### 3.3 CBD Inhibits Aberrant Activation and Inflammatory Responses of BBB Astrocytes in LPS‐Induced Models

**FIGURE 3:**
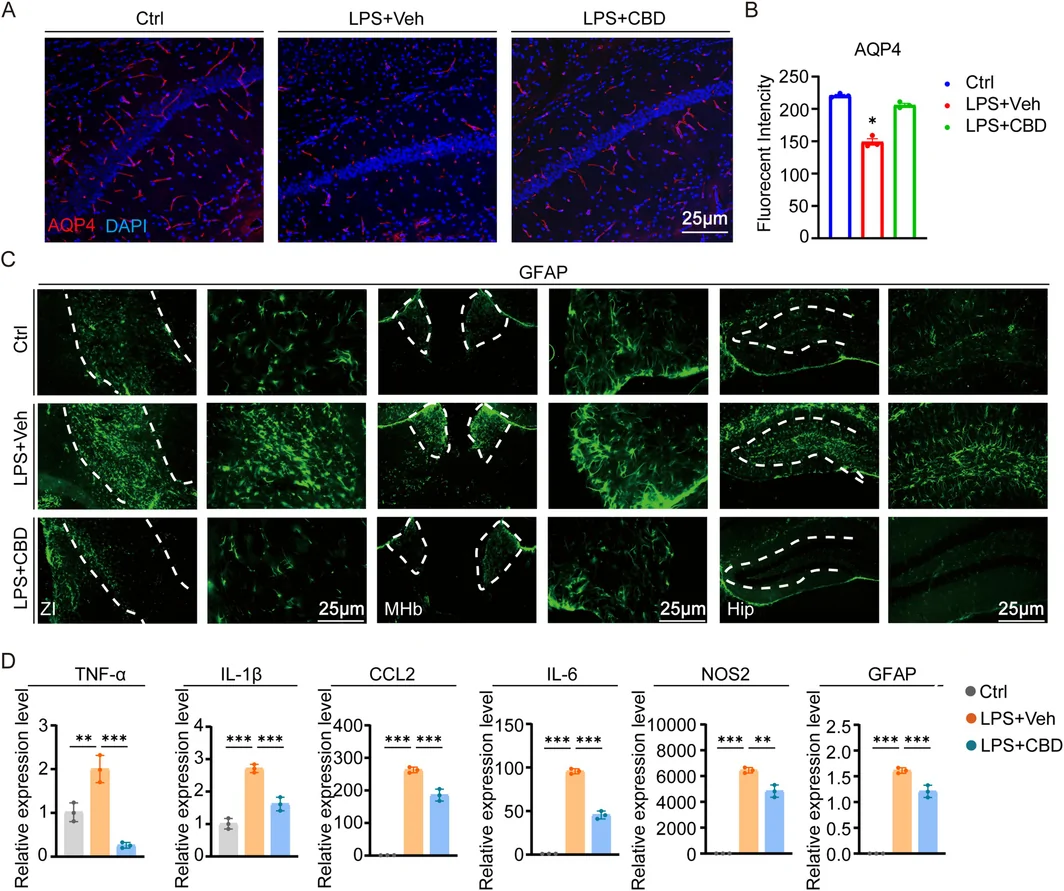
CBD Inhibits Aberrant Activation and Inflammatory Responses of BBB Astrocytes in LPS‐Induced Models. (A) Immunofluorescence staining of AQP4 in hippocampal sections. Scale bar: 25 μm. (B) Quantitative analysis of AQP4 in (A). (C) GFAP immunofluorescence staining in the hippocampus, medial habenula, and septum pallucidum of mice from the three groups. (D) Expression levels of GFAP and pro‐inflammatory cytokines (TNF‐α, IL‐1β, IL‐6, CCL2, and NOS2) in the hippocampus of the three groups of mice. Data are presented as mean ± SEM (*n* = 3). One‐way ANOVA with Tukey's post hoc test was used for statistical analysis; ns, not significant; *p < 0.05, **p < 0.01, ***p < 0.001.

Astrocytes recruited at a later stage further assist endothelium in acquiring BBB characteristics, barrier properties, and CNS immune quiescence [25]. Thus, we evaluated the impact of depression on astrocytes. Aquaporin‐4 (AQP4) is highly polarized to the perivascular astrocyte membrane, where it plays a critical role in maintaining blood–brain barrier (BBB) integrity by facilitating water transport between cerebrospinal fluid and interstitial fluid [26]. Quantitative analysis revealed that mice in the LPS group exhibited a significant reduction in AQP4 compared to controls, indicating a decrease in AQP4‐positive astrocytes. This change reflects LPS‐induced astrocyte inflammatory injury, and the reduction was reversed by CBD treatment (Figure 3A,B). Moreover, we observed more GFAP^+^ cells in the Hip, MHb, ZI of the LPS group (Figure 3C). Real‐time qPCR results were further confirmed with IF. Again, the mRNA expression of GFAP and its activation‐related proinflammatory cytokines (TNF‐α, IL‐1β, IL‐6, CCL2 and NOS2) in the Hip was elevated in the LPS group (Figure 3D). ELISA analysis of mouse serum revealed that the LPS group exhibited approximately two‐fold increases in TNF‐α, IL‐1β, and IL‐6 levels compared to the control group (Figure S1C), a magnitude far below the 10‐fold or higher elevations typically associated with sepsis, confirming that our model represents a low‐grade, non‐lethal inflammatory state rather than systemic sepsis. Above all, CBD treatment inhibits astrocytes' aberrant activation and inflammatory response, indicating its protective effect on BBB.

### 3.4 CBD Improves Integrity of co‐Cultured Blood–Brain Barriers in LPS‐Induced Damage

**FIGURE 4:**
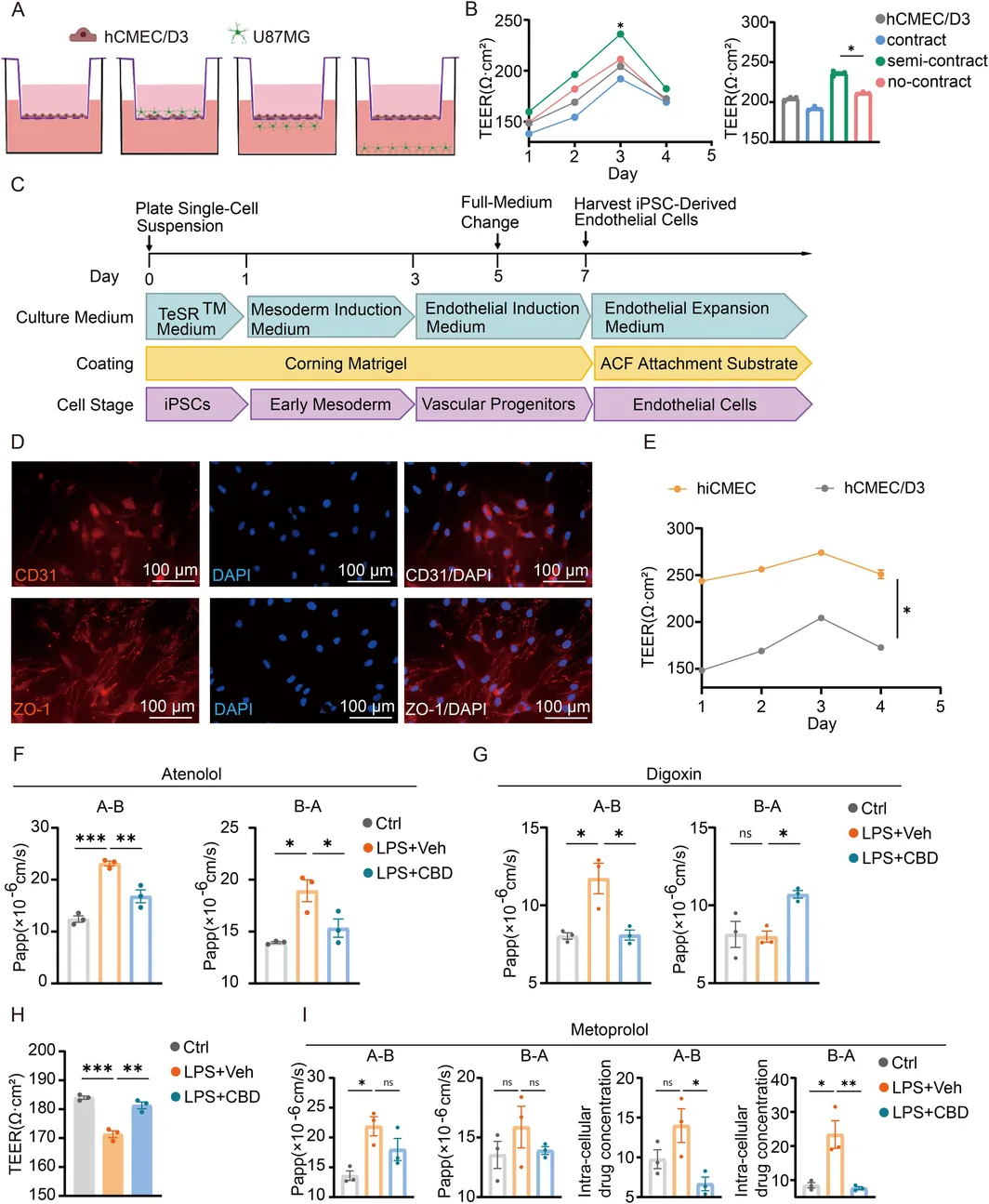
CBD improves integrity of co‐cultured blood–brain barriers in LPS‐induced damage. (A) Schematic diagram of four kinds of BBB model in vitro consisting of hCMEC/D3 cells, direct contact co‐culture, indirect contact co‐culture and non‐contact co‐culture. (B) TEER of four kinds of BBB model in vitro at different time points. (C) Schematic diagram of hiCMECs differentiated from iPSCs. (D) Immunofluorescence staining of endothelial marker CD31 in hiCMECs. (E) TEER values of hiCMEC–U87MG and hCMEC/D3–U87MG co‐culture models. Detection of drug membrane permeability in (F) Atenolol, (G) Digoxin and (I) Metoprolol in the apical‐to‐basolateral and basolateral‐to‐apical directions. (H) The value of TEER in three groups of indirect contact hiCMEC–U87MG co‐culture model. Data are presented as mean ± SEM (*n* = 3). One‐way ANOVA with Tukey's post hoc test was used for statistical analysis; ns, not significant; *p < 0.05, **p < 0.01, ***p < 0.001.

To further verify the protective effect of CBD on the BBB, we established co‐cultured BBB models composed of hCMEC/D3 and U87MG using direct contact, indirect contact, and non‐contact co‐culture methods for inoculation in vitro (Figure 4A) [27]. The TEER value increased with time due to the confluency of cells, which showed peak values 3 days post‐seeding, and the indirect contact co‐culture transwell showed maximum resistance compared to other transwells (Figure 4B). To improve the quality of the co‐culture BBB model, iPSCs were then differentiated to hiCMEC (Figure 4C). As evidenced by the expression of the iPSCs markers NANOG, POUSF1, and SOX2, the mesoderm markers SHH and FGF8, and endothelial cell markers CD31, ZO‐1, and Claudin‐5 (Figure S1C,F,G), iPSCs' fate was successfully induced in cerebral microvascular endothelial cells (hiCMEC) (Figure 4D). The co‐cultured (hiCMEC and U87MG) transwell showed a greater TEER value than the co‐cultured (hCMEC/D3 and U87MG) transwell (Figure 4E). The TEER of the co‐cultured (hiCMEC and U87MG) transwell was significantly decreased after treatment with LPS, and this decrease was significantly ameliorated by CBD (10 μmol/L) (Figure 4H). Transcytosis was assessed in both apical‐to‐basal and basal‐to‐apical directions. In accordance with the TEER values, the decreased atenolol, metoprolol, and digoxin signals of co‐cultured (hiCMEC and U87MG) transwell were observed in the CBD group compared to the LPS group (Figure 4F,G,I). These results indicated that CBD could increase the TEER and decrease paracellular permeability in LPS‐induced blood brain barrier damage.

### 3.5 CBD Inhibits ACLY ^+^p300^+^ Epigenetic Memory Astrocytes Activation and Alleviates Depressive‐Like Behaviors in Mice

**FIGURE 5:**
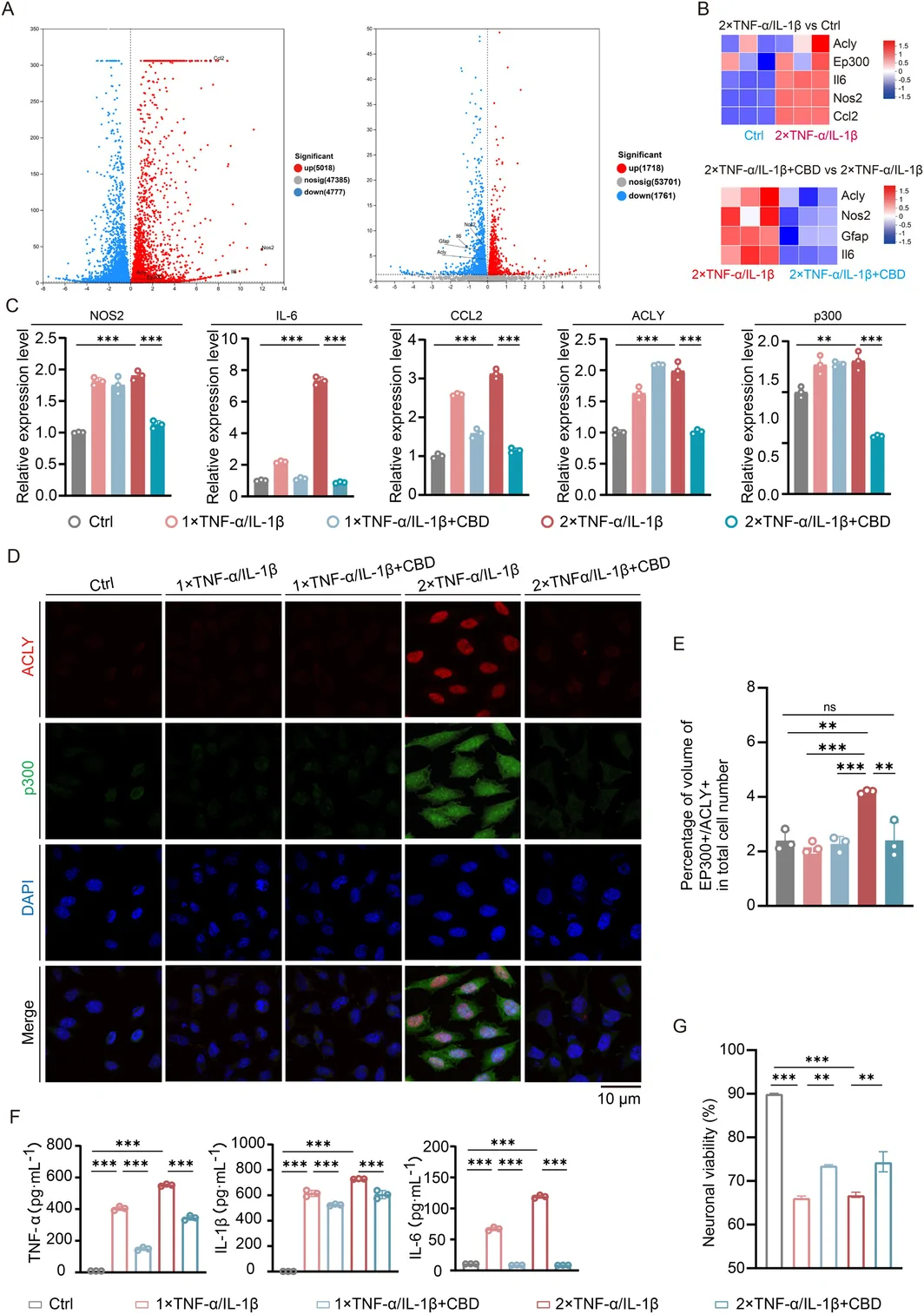
CBD inhibits ACLY^+^p300^+^ epigenetic memory astrocytes activation and attenuates their exacerbated pro‐inflammatory responses. (A) Transcriptomic volcano plots (left: 2 × TNF‐α/IL‐1β vs. control; right: 2 × TNF‐α/IL‐1β + CBD vs. 2 × TNF‐α/IL‐1β). (B) Heatmaps corresponding to panel A, highlighting representative genes (top: Acly, Ep300, Il6, Nos2, Ccl2; bottom: Nos2, Acly, Il6, Gfap). (C) qRT‐PCR analysis of pro‐inflammatory genes (Nos2, Il6, Ccl2, Acly, Ep300) across five groups. (D) Immunofluorescence staining of p300 and ACLY in astrocytes from five groups. (E) Quantification of p300 and ACLY immunofluorescence signals shown in panel D. (F) ELISA analysis of TNF‐α, IL‐1β, and IL6 levels across five groups. (G) Neuronal viability assay. Data are presented as mean ± SEM (*n* = 3). One‐way ANOVA with Tukey's post hoc test was used for statistical analysis; ns, not significant; **p < 0.01, ***p < 0.001.

**FIGURE 6:**
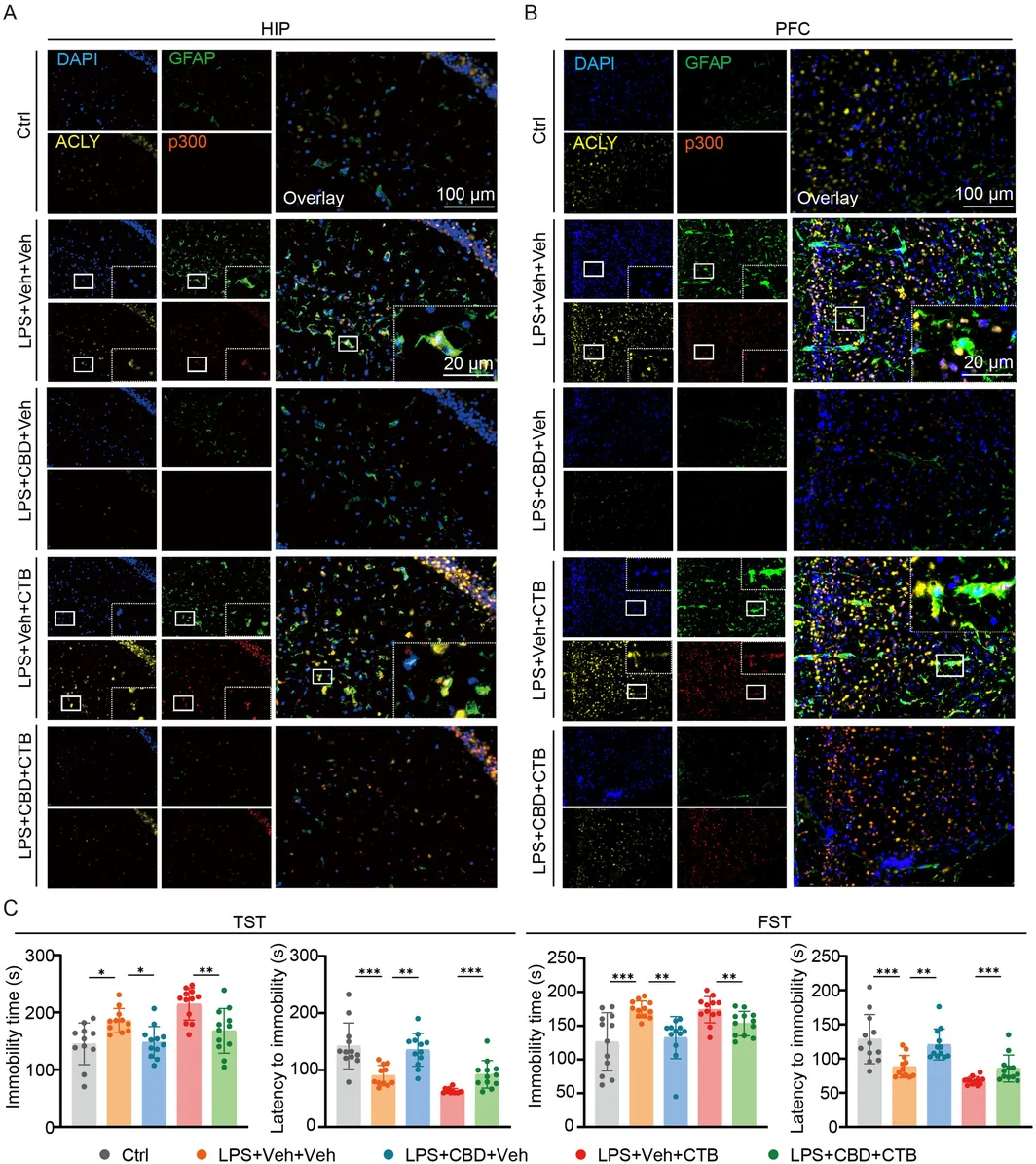
CBD inhibits ACLY^+^p300^+^ epigenetic memory astrocytes activation and alleviates depressive‐like behaviors in mice immunofluorescence staining of GFAP, p300 and ACLY in the (A) HIP and (B) PFC of mice from the five groups, *n* = 3. (C) Immobility time and latency to immobility in the TST and FST, Data are presented as mean ± SEM (*n* = 12). One‐way ANOVA with Tukey's post hoc test was used for statistical analysis; ns, not significant; *p < 0.05, **p < 0.01, ***p < 0.001.

The epigenetic memory astrocyte subset (ACLY^+^p300^+^) displays faster and stronger pro‐inflammatory responses upon restimulation and compromises the integrity and function of BBB. Therefore, we investigated the effect of CBD on the memory astrocyte subset. We stimulated primary mouse brain astrocytes two times 1 week apart (2 × TNF‐α/IL‐1β), which were first identified by GFAP immunofluorescence staining (Figure S1H) and next conducted RNA transcriptome sequencing. We identified the differentially expressed genes (DEGs) in the Ctrl, 2 × TNF‐α/IL‐1β and 2 × TNF‐α/IL‐1β + CBD groups. There was an increase in the expression of ACLY, p300, IL‐6, NOS2 and CCL2 in the 2 × TNF‐α/IL‐1β group compared with those in the Ctrl group. Additionally, the expression of ACLY, NOS2, IL‐6 and GFAP decreased in the 2 × TNF‐α/IL‐1β + CBD group compared with the 2 × TNF‐α/IL‐1β group (Figure 5A,B). Consistently, compared with stimulation with 1 × TNF‐α/IL‐1β alone, ACLY^+^p300^+^ astrocytes significantly upregulated the expression of genes associated with astrocyte pro‐inflammatory activities, including IL‐6, CCL2, and NOS2 (Figure 5C–E), while reducing neuronal viability (Figure 5G). Notably, CBD not only inhibited the formation of ACLY^+^p300^+^ astrocytes but also attenuated the exacerbated inflammatory responses (as reflected by reduced levels of TNF‐α, IL‐1β, and IL‐6) induced by restimulation (Figure 5F). Indeed, we detected a marked increase in the number of ACLY^+^p300^+^ astrocytes in the Hip and PFC of mice exhibiting depressive‐like behaviors (Figure 6A,B). It is worth noting that CTB, an agonist of p300, promotes the generation of memory astrocytes; in contrast, CBD exerts a distinct inhibitory effect on this process and significantly alleviates depressive‐like behaviors in mice (Figure 6C). Collectively, these findings provide a solid experimental basis and a clear research direction for the development of therapeutic strategies against depression.

## 4 Discussion

Depression is increasingly recognized as a multifactorial disorder characterized by complex interactions between neuroinflammation and blood–brain barrier (BBB) impairment [28, 29]. Specifically, proinflammatory cytokines derived from neuroinflammation (e.g., TNF‐α, IL‐1β) can disrupt BBB integrity through the degradation of tight junction proteins, whereas BBB leakage, in turn, exacerbates neuroinflammation by permitting the infiltration of peripheral immune cells and harmful substances into the central nervous system, thereby collectively contributing to the progression of depressive pathology [30, 31]. Consequently, restoring BBB integrity and inhibiting neuroinflammation have been proposed as critical therapeutic strategies for depression. Notably, CBD is well‐documented to exert antidepressant, anti‐inflammatory, and neuroprotective effects [32, 33, 34]. The optimal dosage of CBD has been documented in prior investigations. Numerous studies have indicated that 30 mg/kg of CBD exhibits favorable therapeutic efficacy [35, 36]. More importantly, our data show that a single administration of CBD effectively alleviated depressive‐like behaviors, including reduced exploratory activity and increased despair‐like behavior (Figure 1). However, it is important to acknowledge the limitations of this acute/short‐term observation paradigm, and we recognize that long‐term efficacy, safety, and tolerability assessments are needed to fully evaluate the translational potential of CBD for CNS disorders. Taken together, these observations suggest that our study may provide an effective strategy for the treatment of depression.

The blood–brain barrier, a specialized barrier formed by brain capillary endothelial cells, pericytes, astrocytes, and basement membrane, regulates blood–brain substance transport to protect the central nervous system and maintain its homeostasis [37]. When the body is subjected to stress (such as social stress or restraint stress) or suffers from brain injury, the integrity of the BBB will be significantly damaged: the expression level of tight junction proteins in brain microvascular endothelial cells decreases, directly leading to an abnormal increase in BBB permeability [38, 39, 40]. This structural damage can further induce reactive astrocyte activation, and the activated astrocytes may exacerbate the damage to the CNS through cascade reactions. Notably, reactive astrocytes secrete substances such as vascular endothelial growth factor and matrix metalloproteinase 9 (MMP9) during activation [41]. In addition, regulating the abnormal activation of reactive astrocytes can promote the production of neurotrophic and vascular protective factors such as brain‐derived neurotrophic factor (BDNF), fatty acid‐binding proteins, and angiopoietins [15]. These factors participate in the repair process of the BBB through mechanisms such as enhancing endothelial cell function and promoting vascular repair, thereby providing support for the recovery of CNS function. Concerning the improvement of BBB permeability by CBD, previous studies have validated this in models of multiple sclerosis, sepsis, stroke [42] and traumatic brain injury (TBI) [43]. Our results indicate that CBD stabilizes tight junction complexes and preserves endothelial barrier integrity (Figure 2). This effect limits the infiltration of peripheral pro‐inflammatory mediators into the CNS and mitigates neuroinflammatory cascades that drive depressive‐like behaviors (Figure 3).

The present study demonstrates that a single dose of cannabidiol (CBD) exerts protective effects on the blood–brain barrier (BBB) and alleviates depressive‐like behaviors, with its mechanisms potentially linked to the regulation of memory astrocytes and their mediated neuroinflammation. These findings provide novel insights into the pathophysiological interplay between BBB integrity, neuroinflammation, and depression, while highlighting CBD as a promising therapeutic candidate for depressive disorders.

BBB dysfunction is increasingly recognized as a key pathological feature of depression, with endothelial tight junction disruption and neuroinflammatory infiltration contributing to mood dysregulation. Our results confirm that CBD ameliorates the downregulation of endothelial tight junction proteins induced by LPS, consistent with previous reports of its BBB‐protective properties. Notably, this effect was attenuated by WAY100135 (a 5‐HT1A receptor antagonist) and GW9662 (a PPARγ antagonist), reinforcing that CBD acts through 5‐HT1A and PPARγ receptors to stabilize BBB structure—providing direct evidence for receptor‐mediated regulatory pathways of CBD on BBB integrity [44, 45]. Additionally, we also observed the protective effect of CBD on endothelial cells in vitro, which is in accordance with the findings in the literature. To further examine endothelial‐specific responses under controlled conditions, we employed in vitro BBB models based on human endothelial cells. The hCMEC/D3 cell line was initially used as a stable and widely adopted screening model, whereas iPSC‐derived endothelial cells were subsequently introduced to construct a BBB model with improved barrier properties and higher physiological relevance. Consistent with the in vivo findings, CBD increased TEER values and reduced transendothelial permeability in the LPS‐treated co‐culture systems.

A key novel finding of this study is the identification of memory astrocytes as a critical target of CBD in this process. Our data indicate that CBD not only alleviates depressive‐like behaviors and restores BBB integrity, but also significantly suppresses the accumulation of ACLY^+^p300^+^ memory astrocytes in the brain and their associated exaggerated inflammatory responses. Memory astrocytes, characterized by epigenetic priming after initial inflammatory stimulation, exhibit exacerbated pro‐inflammatory responses upon re‐stimulation via the ACLY‐p300 axis: ACLY‐derived acetyl‐CoA promotes p300‐mediated histone acetylation, maintaining chromatin accessibility at pro‐inflammatory gene loci [46, 47]; subsequent activation of NF‐κB signaling [48] upregulates cytokines such as IL‐6, CCL2, and NOS2 [49], while impairing neuronal metabolic support (e.g., reduced lactate release). The brain displays the highest p300 and CBP HAT activity in the body [50]. p300 regulates cellular senescence and stress response, which promote systemic chronic inflammation [51, 52]. This persistent pro‐inflammatory phenotype may exacerbates chronic central nervous system (CNS) inflammation and drives BBB disruption—linking neuroinflammation to BBB damage in depression. Based on our observations that CBD downregulates the expression of ACLY, p300, and key pro‐inflammatory mediators, it is likely that CBD alleviates the generation or reactivity of memory astrocytes by suppressing this ACLY‐p300 epigenetic amplifier module. This intervention may in turn reduce secondary neuroinflammatory damage, thereby supporting blood–brain barrier integrity and alleviating depressive‐like behaviors (Figures 5 and 6). This aligns with emerging evidence that targeting disease‐associated astrocyte subsets can mitigate CNS pathology in chronic neuroinflammatory disorders [16]. We acknowledge that the current evidence primarily establishes an association at the expression level, future studies employing functional genetics are needed to definitively establish causality within this proposed axis and to fully delineate its interplay with canonical inflammatory pathways.

Despite these insights, the specific molecular mechanisms underlying the interaction between CBD and memory astrocytes remain to be fully elucidated. Two potential pathways warrant further investigation: First, CBD may act directly on memory astrocytes via specific receptors (e.g., 5‐HT1A or PPARγ, which are expressed on astrocytes) to suppress ACLY‐p300‐dependent epigenetic programming, thereby reducing their pro‐inflammatory potential. Second, CBD could indirectly regulate memory astrocyte function by stabilizing other BBB components (e.g., pericytes or endothelial cells), as bidirectional communication between astrocytes and BBB‐resident cells is critical for maintaining CNS homeostasis. Disentangling these possibilities will require cell‐type‐specific manipulation studies, such as conditional knockout of 5‐HT1A or PPARγ in astrocytes, or co‐culture models to assess crosstalk between endothelial cells and memory astrocytes.

In this study, we relied on GFAP as a single astrocytic marker. Given that the memory astrocyte subset is defined by the co‐expression of GFAP, ACLY, and EP300, whether other astrocytic markers can label the same subset remains unclear and requires further investigation. Future studies using single‐cell RNA sequencing of astrocytes from in vivo models are warranted to validate the heterogeneity of memory astrocyte subsets and to explore additional subset‐specific markers. Currently, our evidence is largely in vitro, and future work should address these limitations.

In conclusion, our study identifies a novel role for memory astrocytes in linking neuroinflammation, BBB dysfunction, and depression, and highlights CBD as a potential therapeutic agent that targets this axis. These findings not only expand our understanding of the pathophysiology of depression but also provide a rationale for future research into CBD‐based interventions, with a focus on clarifying its precise mechanisms of action on memory astrocytes. Given the unmet clinical need for novel antidepressants with neuroprotective properties, CBD merits further investigation as a candidate for mitigating BBB damage and depressive symptoms through the regulation of pathological astrocyte subsets.

## Author Contributions

Experimental design and article writing: Yue Chen, Hui Ma, Qian Long and Xuan Fang. Experimental operation and data analysis: Yue Chen, Hui Ma, Qian Long, Xuan Fang, Xiaotong Li, Wenhui Xiao, Xinzhou Deng, Fuze Du, Yifei Tan. Guidance for the experimental design and data analysis: Xin Qiao, Xiaoxia Jiang and Bo Gao. Article revision: Xin Qiao, Xiaoxia Jiang, Yan Wang and Bo Gao. All authors have seen and approved the final version of the manuscript being submitted. We warrant that the article is the authors' original work, hasn't received prior publication and isn't under consideration for publication elsewhere.

## Funding

This work was supported by the Natural Science Foundation of Beijing Municipality (7232348, 7242278) and the National Natural Science Foundation of China (82204360).

## Ethics Statement

All the procedures were approved by the Institutional Animal Care and Use Committee of Academy of Military Medical Sciences, Academy of Military Sciences (JFALR2018098). All animal studies are reported in compliance with the ARRIVE guidelines.

## Conflicts of Interest

The authors declare no conflicts of interest.

## Supporting information


**Figure S1:** CBD alleviates LPS‐induced systemic inflammation, blood–brain barrier disruption, and astrocyte activation in vivo, with characterization of iPSC and primary astrocyte cultures. (A) Total distance traveled in the open field test, *n* = 8. Data represent the mean ± SEM; ns, not significant, ***p < 0.001. (B) Body weight records during the 7‐day modeling period. Body weight was measured daily in the Ctrl, LPS + Veh, and LPS + CBD groups. (C) Serum levels of pro‐inflammatory cytokines (TNF‐α, IL‐1β, and IL‐6) in the three groups. Cytokine concentrations were measured by ELISA, *n* = 3. Data represent the mean ± SEM; ***p < 0.001. (D) Representative brain images showing FITC‐dextran extravasation of the three groups. (E) Representative immunofluorescence images of tight junction proteins (claudin‐5, Occludin, and ZO‐1) in the three groups. Scale bar = 100 or 25 μm. (F) Immunofluorescence staining of pluripotency marker NANOG in iPSCs. Scale bar = 200 or 100 μm. (G) Quantitative RT‐PCR of the expression of markers in induction process, *n* = 3. Data represent the mean ± SEM; ns, not significant; *p < 0.05, **p < 0.01 and ***p < 0.001. (H) Representative immunofluorescence images of primary astrocytes identified by GFAP staining. The images confirm the purity and morphology of the astrocyte culture. Scale bar = 20 μm.

## Data Availability

The datasets generated during and/or analyzed during the current study are available from the corresponding author on reasonable request.
