# Supplementary material for: Cannabidiol Ameliorates Blood–Brain Barrier Dysfunction and Inhibits Memory Astrocytes Activation in Mice With Sickness‐Like Behaviors
Source: CNS Neurosci Ther. 2026 Sep 16;32(9):e71168. doi: 10.1002/cns.71168 (PMC13580345; doi:10.1002/cns.71168)
Supplement: Supplementary file 1 [file CNS-32-e71168-s001.docx]

**Supplementary** **Fig.1** **CBD alleviates LPS-induced systemic inflammation, blood-brain barrier disruption, and astrocyte activation in vivo, with characterization of iPSC and primary astrocyte cultures.** **A) Total distance traveled in the open field test, n=8.** Data represent the mean ± SEM; ns, not significant, ***p<0.001. B) Body weight records during the 7‑day modeling period. Body weight was measured daily in the Ctrl, LPS+Veh, and LPS+CBD groups. C) Serum levels of pro-inflammatory cytokines (TNF‑α, IL‑1β, and IL‑6) in the three groups. Cytokine concentrations were measured by ELISA, n=3. Data represent the mean ± SEM; ***p<0.001. D) **Representative brain images showing FITC-dextran extravasation of the three groups.** E) Representative immunofluorescence images of tight junction proteins (claudin-5, Occludin, and ZO-1) in the three groups. Scale bar = 100 or 25 µm. F) Immunofluorescence staining of pluripotency marker NANOG in iPSCs. Scale bar = 200 or 100 µm. G) Quantitative RT-PCR of the expression of markers in induction process, n=3. Data represent the mean ± SEM; ns, not significant; *p<0.05, **p < 0.01 and ***p<0.001. H) Representative immunofluorescence images of primary astrocytes identified by GFAP staining. The images confirm the purity and morphology of the astrocyte culture. Scale bar =20 µm.
